# S100P在肺腺癌中的表达及其临床意义

**DOI:** 10.3779/j.issn.1009-3419.2026.101.12

**Published:** 2026-05-20

**Authors:** Ziyi DING, Donghui ZHANG, Nanyang LI, Kai LUO, Jiahao CHEN, Binghui DING, Qian WANG

**Affiliations:** 1 510000 广州，广州医科大学附属肿瘤医院病理科; 1 Department of Pathology, Guangzhou Institute of Cancer Research, The Affiliated Cancer Hospital,Guangzhou Medical University, Guangzhou 510000, China; 2 510000 广州，广州医科大学附属肿瘤医院检验科; 2 Department of Clinical Laboratory, Guangzhou Institute of Cancer Research, The Affiliated Cancer Hospital,Guangzhou Medical University, Guangzhou 510000, China; 3 510000 广州，广州医科大学附属肿瘤医院肝胆外科; 3 Department of Hepatobiliary Surgery, Guangzhou Institute of Cancer Research, The Affiliated Cancer Hospital,Guangzhou Medical University, Guangzhou 510000, China; 4 510000 广州，广州医科大学附属肿瘤医院胸外科; 4 Department of Thoracic Surgery, Guangzhou Institute of Cancer Research, The Affiliated Cancer Hospital,Guangzhou Medical University, Guangzhou 510000, China

**Keywords:** 肺肿瘤, S100P, 生物标志物, 预测潜力, Lung neoplasms, S100P, Biomarkers, Predictive potential

## Abstract

**背景与目的 **肺腺癌（lung adenocarcinoma, LUAD）是非小细胞肺癌最常见的组织学亚型。由于早期无明显症状及体征，多数患者就诊时处于中晚期，治疗困难，预后较差。因此，筛选鉴定高效、特异的肺癌生物标志物仍是研究热点。本研究旨在探究S100钙结合蛋白P（S100 calcium-binding protein P, S100P）在LUAD中的表达及其临床意义。**方法** 收集广州医科大学附属肿瘤医院LUAD组织、癌旁正常组织和肺炎症性病变组织各50例分别作为LUAD组、正常组和良性组，利用免疫组织化学SP法检测钙结合蛋白家族成员S100P、C-C趋化因子配体4（C-C chemokine ligand 4, CCL4）及白细胞跨膜糖蛋白8（cluster of differentiation 8, CD8）在相应组织中的表达情况并进行相关性分析。**结果** 基于UALCAN和GEPIA2数据库，分别获得了相对正常组织的LUAD mRNA高表达差异基因谱。免疫组化结果显示S100P蛋白、CCL4蛋白在临床LUAD组织中的表达均高于对照组以及良性组（*P*均<0.05）。S100P蛋白表达用于辅助鉴别LUAD的曲线下面积可达0.943。S100P蛋白表达与LUAD患者的年龄、性别、肿瘤大小及分化程度均无明显关联（*P*均>0.05）。相关性分析显示，S100P蛋白与CCL4蛋白表达呈中度正相关（*r*=0.611, *P*<0.001），S100P蛋白表达与CD8^+^淋巴细胞浸润呈正相关（*r*=0.461, *P*<0.001），CCL4蛋白表达与CD8^+^淋巴细胞浸润也呈正相关（*r*=0.400, *P*<0.001）。**结论** 数据库分析显示，S100P在LUAD中高表达且与不良预后有关，是LUAD预后相关生物标志物。同时，临床组织分析发现S100P高表达还与LUAD中CD8^+^淋巴细胞浸润相关，S100P是LUAD淋巴细胞浸润的生物标志物，并具有预测免疫检查点抑制剂疗效的潜力。

## Abstract (en)

**Backgroud and objective** Lung adenocarcinoma (LUAD) is the most common histological subtype of non-small cell lung cancer (NSCLC). Due to the lack of obvious symptoms and signs in the early stages, most patients are already in the middle or late stages at diagnosis. This makes treatment difficult and prognosis poor. Therefore, the identification of effective and specific biomarkers for lung cancer remains a focal area of research. So this study aimed to investigate the expression and clinical significance of S100 calcium-binding protein P (S100P) in LUAD. **Methods** Tissue samples of LUAD (*n*=50), adjacent normal lung tissue (*n*=50), and benign inflammatory lesions (*n*=50) were collected from The Affiliated Cancer Hospital, Guangzhou Medical University, used as the LUAD group, normal group and benign group. Immunohistochemistry (SP method) was employed to detect the expression of S100P, C-C chemokine ligand 4 (CCL4), and cluster of differentiation 8 (CD8) proteins. Correlation analyses were carried out. **Results** Database analysis (UALCAN, GEPIA2) identified S100P as an mRNA with high expression in LUAD. Immunohistochemistry demonstrated that the protein levels of S100P and CCL4 were significantly higher in lung tissues of the LUAD group compared to those in the normal group and benign group (all *P*<0.05). The area under the curve (AUC) of S100P for differentiating LUAD was 0.943. The expression of S100P showed no significant correlation with age, sex, tumor size, or degree of differentiation (all *P*>0.05). Correlation analysis revealed that S100P expression was moderately positively correlated with CCL4 (*r*=0.611, *P*<0.001), and positively correlated with CD8^+^ lymphocyte infiltration (*r*=0.461, *P*<0.001). CCL4 expression was also positively correlated with CD8^+^ infiltration (*r*=0.400, *P*<0.001). **Conclusion** Database analysis indicates that S100P is highly expressed in LUAD and associated with a poor prognosis, making it a potential biomarker for the prognosis of LUAD. Clinical tissue analysis further shows that S100P expression is correlated with CD8^+^ lymphocyte infiltration in LUAD, suggesting that S100P can serve as a biomarker of lymphocyte infiltration and a potential predictor of the response to immune checkpoint inhibitors.

肺癌是全球发病率和死亡率最高的恶性肿瘤之一，严重威胁人类健康。肺癌包括小细胞肺癌和非小细胞肺癌（non-small cell lung cancer, NSCLC），其中NSCLC是肺癌的主要类型，占比85%^[1]^，而肺腺癌（lung adenocarcinoma, LUAD）是NSCLC最常见的组织学亚型。由于早期无明显症状及体征，多数患者就诊时处于中晚期，治疗困难，预后较差。因此，筛选鉴定高效、特异的肺癌生物标志物仍是研究热点。理想的标志物不仅有助于早期诊断和预后判断，更可为个体化诊疗策略的制定提供关键依据。

近年来，NSCLC的治疗取得了突破性进展。以免疫检查点抑制剂（immune checkpoint inhibitors, ICIs）为代表的免疫治疗已成为NSCLC的重要治疗手段之一。相关研究^[2]^显示，程序性死亡受体1（programmed cell death 1, PD-1）抑制剂西米普利（Cemiplimab）单药治疗程序性死亡配体1（programmed cell death ligand 1, PD-L1）高表达（≥50%）患者可显著改善无进展生存期和总生存期，其与化疗联合方案在PD-L1≥1%患者中同样生存获益。然而，免疫治疗的疗效存在显著的个体差异性，目前已有的疗效预测标志物如PD-L1表达和肿瘤突变负荷（tumor mutational burden, TMB）均存在一定的局限性，如异质性高、影响因素多、预测效果待进一步提高等；需结合微卫星不稳定性（microsatellite instability, MSI）、免疫微环境特征等多维度指标，或采用动态监测、多组学分析等方法，以提高预测准确性。因此，探寻更多、可靠的免疫治疗疗效预测标志物，对于优化免疫治疗策略、精准筛选获益人群具有重要的临床意义。Sun等^[3]^研究发现通过比较NSCLC组织与正常组织，共发现6778个差异表达基因（P<0.05），其中490个为差异性免疫相关基因。生存分析确定28个与预后相关的差异免疫基因（P<0.05）；绘制风险曲线后，发现S100钙结合蛋白（S100 calcium-binding protein A16, S100A16）、免疫球蛋白K可变区4（immunoglobulin kappa variable 4, IGKV4）、S100钙结合蛋白P（S100 calcium-binding protein P, S100P）、血管生成素样蛋白4（angiopoietin-like protein 4, ANGPTL4）、信号素4B（semaphorin 4B, SEMA4B）和富含亮氨酸重复序列的G蛋白偶联受体4（leucine-rich repeat-containing G-protein-coupled receptor 4, LGR4）为NSCLC的高风险免疫基因。Yu等^[4]^研究发现LUAD共鉴定出10个药物耐药正相关基因血小板白细胞C激酶底物2（pleckstrin 2, PLEK2）、转录因子激活蛋白-2α（transcription factor AP-2 alpha, TFAP2A）、驱动蛋白家族成员20A（kinesin family member 20A, KIF20A）、S100P、生长分化因子15（growth differentiation factor 15, GDF15）、小分子热休克蛋白B家族成员8（heat shock protein family B member 8, HSPB8）、SAM和SH3结构域包含蛋白1（SAM and SH3 domain containing 1, SASH1）、Wiskott-Aldrich综合征蛋白家族成员3（Wiskott-Aldrich syndrome protein family, member 3, WASF3）、层粘连蛋白α3亚基（laminin subunit alpha 3, LAMA3）和转钴胺素蛋白1（transcobalamin 1, TCN1）。由这10个基因构建的LUAD风险评分模型能够可靠预测LUAD患者的预后。

本研究以LUAD为研究对象，首先利用公共数据库筛选获得LUAD标志物S100P，然后进一步分析了S100P与相关分子在介导肿瘤免疫浸润及预测免疫治疗疗效中的作用，最后利用临床样本进行了实验验证，为阐明S100P作为LUAD标志物和潜在免疫治疗疗效预测标志物的作用提供了实验依据。

## 1 材料与方法

### 1.1 UALCAN分析

UALCAN数据库（https://ualcan.path.uab.edu/）是一个癌症数据分析门户网站，集成了多个大型癌症研究项目数据，可进行癌症差异基因表达分析、生存分析、泛癌分析和关联分析等。本研究首先通过分析数据库癌症差异基因表达数据，获得LUAD高表达基因谱，通过与GEPIA2数据库获得的LUAD高表达基因谱取交集，得到LUAD相关候选基因列表。然后，通过对高表达排序前10的基因做进一步表达分析、生存分析和泛癌分析筛选出具有潜在临床价值的LUAD相关基因S100P。最后，利用数据库进行S100P与临床特征的相关分析。

### 1.2 GEPIA2分析

GEPIA2数据库（http://gepia2.cancer-pku.cn/）是由北京大学开发的，基于癌症基因组图谱（The Cancer Genome Atlas, TCGA）和基因型-组织表达（Genotype-Tissue Expression, GTEx）数据的癌症数据在线分析网站，可执行癌症基因差异表达分析、生存分析、相关性分析、转录本详情查询等功能。本研究利用该数据库进行了LUAD差异基因表达分析、LUAD相关候选基因生存分析。

### 1.3 Kaplan-Meier plotter分析

Kaplan-Meier plotter数据库（https://kmplot.com/analysis/）是一个整合了基因表达综合数据库（Gene Expression Omnibus, GEO）、欧洲基因组-表型档案库（European Genome-phenome Archive, EGA）和TCGA等公共数据库中的转录组测序（RNA sequencing, RNA-Seq）和基因芯片数据，专注于肿瘤生存分析的数据库。本研究利用该数据库进一步验证S100P高表达与LUAD患者生存的关系。

### 1.4 验证S100P在正常组织和配对LUAD组织中的表达

从TCGA数据库（https://portal.gdc.cancer.gov）中下载并整理TCGA-LUAD项目RNA-seq数据并提取TPM格式的数据，提取对应编号配对的癌旁和癌样本。

### 1.5 S100P表达与LUAD临床特征的相关性分析

利用UALCAN数据库分析S100P表达与LUAD患者性别、年龄、吸烟、分期等临床特征的相关性。

### 1.6 基因本体论（Gene Ontology, GO）/京都基因与基因组百科全书（Kyoto Encyclopedia of Genes and Genomes, KEGG）通路富集分析

分别利用获取自UALCAN数据库和GEPIA2数据库的LUAD高表达基因谱排名前250位的基因。

### 1.7 基因集癌症分析（Gene Set Cancer Analysis, GSCA）

GSCA数据库（https://guolab.wchscu.cn/GSCA/）是整合了TCGA和抗癌药物敏感性基因组学（Genomics of Drug Sensitivity in Cancer, GDSC）两大权威数据源、覆盖33个癌种的癌症综合数据分析平台，提供基因表达分析、免疫分析、基因组变异分析和药物敏感性分析四大核心分析模块。本研究利用该数据库进行了S100P及相关分子表达与LUAD免疫浸润细胞的相关性分析。

### 1.8 TIMER3分析

TIMER3数据库（https://compbio.cn/timer3/）是一个专注于肿瘤免疫分析的在线综合数据库。本研究利用该数据库进行了S100P基因表达预测免疫治疗疗效的相关分析。

### 1.9 临床样本

收集2019至2024年广州医科大学附属肿瘤医院经手术切除并经病理确诊为LUAD的标本50例作为LUAD组，纳入标准：（1）依据世界卫生组织（World Health Organization, WHO）第5版（2021年）胸部肿瘤分类标准，病理诊断为LUAD；（2）既往无其他恶性肿瘤病史；（3）术前均未进行化疗、放疗、分子靶向药物或免疫治疗等其他抗肿瘤治疗；（4）具有详细完整的病理结果及临床资料。排除标准：（1）确诊LUAD同时合并有如下疾病：糖尿病，冠状动脉粥样硬化性心脏病，房颤，心力衰竭，严重肝、肾功能不全及结缔组织病；为尽可能减少慢性炎症状态及免疫功能异常对相关指标表达的潜在影响，排除合并上述疾病者；（2）临床数据不齐全。其中，男性23例，女性27例，年龄37-79（61.92±9.35）岁，分级不限；50例标本取其相应的癌旁组织（距离肿瘤组织2.0 cm以上）作为正常组。另选取2019至2024年广州医科大学附属肿瘤医院手术切除并经病理学明确诊断为肺炎症性病变标本50例作为良性组，纳入标准：（1）经肺活检获取的肺组织标本，镜下见炎症细胞浸润、肺泡壁增厚、渗出物形成、肺泡上皮细胞变性或坏死；（2）经病理科2名资深病理医生诊断炎症性病变，具体疾病为机化性肺炎、间质性肺炎、炎性假瘤、炎性结节、肉芽肿性炎。排除标准：（1）无典型炎症细胞浸润；（2）无细胞渗出或实变；（3）无间质炎症或纤维化；良性组包括男性29例，女性21例，年龄10-79（55.96±11.69）岁。本研究经过广州医科大学附属肿瘤医院伦理审核（批准文号：GYZL-2024-KY20）。

### 1.10 免疫组织化学检测

采用免疫组织化学SP法。S100P（克隆号：C5B10，批号：202511005）及白细胞跨膜糖蛋白8（cluster of differentiation 8, CD8）（克隆号：C2B11，批号：202512002）抗体购自河南赛诺特生物技术有限公司，C-C趋化因子配体4（C-C chemokine ligand 4, CCL4）（克隆号：多克隆抗体，批号：KB15AA2128）抗体购自无锡傲锐东源生物科技有限公司，二抗及DAB购自丹麦DaKo公司。一抗稀释浓度1:50。将石蜡包埋组织切片（厚度3-4 μm），经二甲苯脱蜡，梯度酒精水化；之后使用pH 9.0 EDTA缓冲液进行高温抗原修复；3%过氧化氢室温孵育阻断内源性过氧化物酶。随后滴加一抗4 ^o^C孵育过夜，次日滴加二抗，常规DAB显色，苏木素复染，经脱水透明后中性树胶封片，光镜下观察；同时设置科室组织芯片为阳性对照；免疫组化结果判定S100P阳性细胞表现为胞核和胞浆呈棕色染色，CCL4阳性细胞表现为胞浆呈棕色染色，CD8阳性细胞表现为胞膜呈棕色染色。结果判定采用2名病理科资深医生双盲独立判定：在高倍镜（×400）下随机选取5个视野取平均值，根据染色强弱和肿瘤阳性细胞比例分数进行综合评分。按染色强度评分：无染色（0分）、弱染色（1分）、中等强度染色（2分）、强染色（3分）；按肿瘤阳性细胞比例分数评分：<1%（0分）、2%-25%（1分）、26%-50%（2分）、51%-75%（3分）、>75%（4分）；总体评分值=肿瘤阳性细胞比例分数评分×染色强度评分。将所有病例乘积评分值分为阴性（-）0-1分、弱阳性（+）2-4分、中等阳性（++）5-8分、强阳性（+++）9-12分；根据总分结果进行定量及定性分析。评分细则及标准参考相关文献^[5,6]^。

### 1.11 统计学分析

采用SPSS 27.0统计软件包进行分析。免疫组织化学评分数据先进行Shapiro-Wilk正态分布检验，如为非正态分布，则以中位数（四分位数）进行数据统计描述，采用非参数检验Kruskal-Wallis H检验S100P、CCL4及CD8的表达水平在LUAD组、正常组及良性组中的差异性，再采用多重假设检验（Dunn’s test）进行两两比较，并通过Bonferroni校正；采用Spearman相关性分析检验评估S100P、CCL4及CD8表达之间的相关性。采用卡方检验分析S100P蛋白表达与LUAD患者临床病理特征的关系，表格中有10个单元格T<5，并行Fisher精确检验精确P值。P<0.05为差异有统计学意义。

## 2 结果

### 2.1 S100P在LUAD中高表达且与不良预后有关

为了筛选新的高特异性LUAD标志物，本研究基于UALCAN数据库获得了相对正常组织的LUAD mRNA高表达差异基因谱（以下简称“高表达差异基因谱”）的排序前250位基因（图1A）。另基于GEPIA2数据库获得了相对正常组织的LUAD mRNA差异基因表达谱（以下简称“差异基因表达谱”）。利用两个不同数据库，结果显示交集共93个基因（图1B）。通过对排序前10位的基因做进一步表达分析、生存分析和泛癌分析，以在多个数据库中mRNA和蛋白层面均高表达且与不良预后有关、同时在多个癌种中呈现高表达为标准，筛出候选LUAD相关基因。结果显示相对正常组，LUAD组S100P基因在mRNA层面均高表达（图1C-1E），差异具有统计学意义。在UALCAN数据库中，LUAD S100P基因在蛋白水平也高表达（图1F），差异具有统计学意义。

**图 1:**
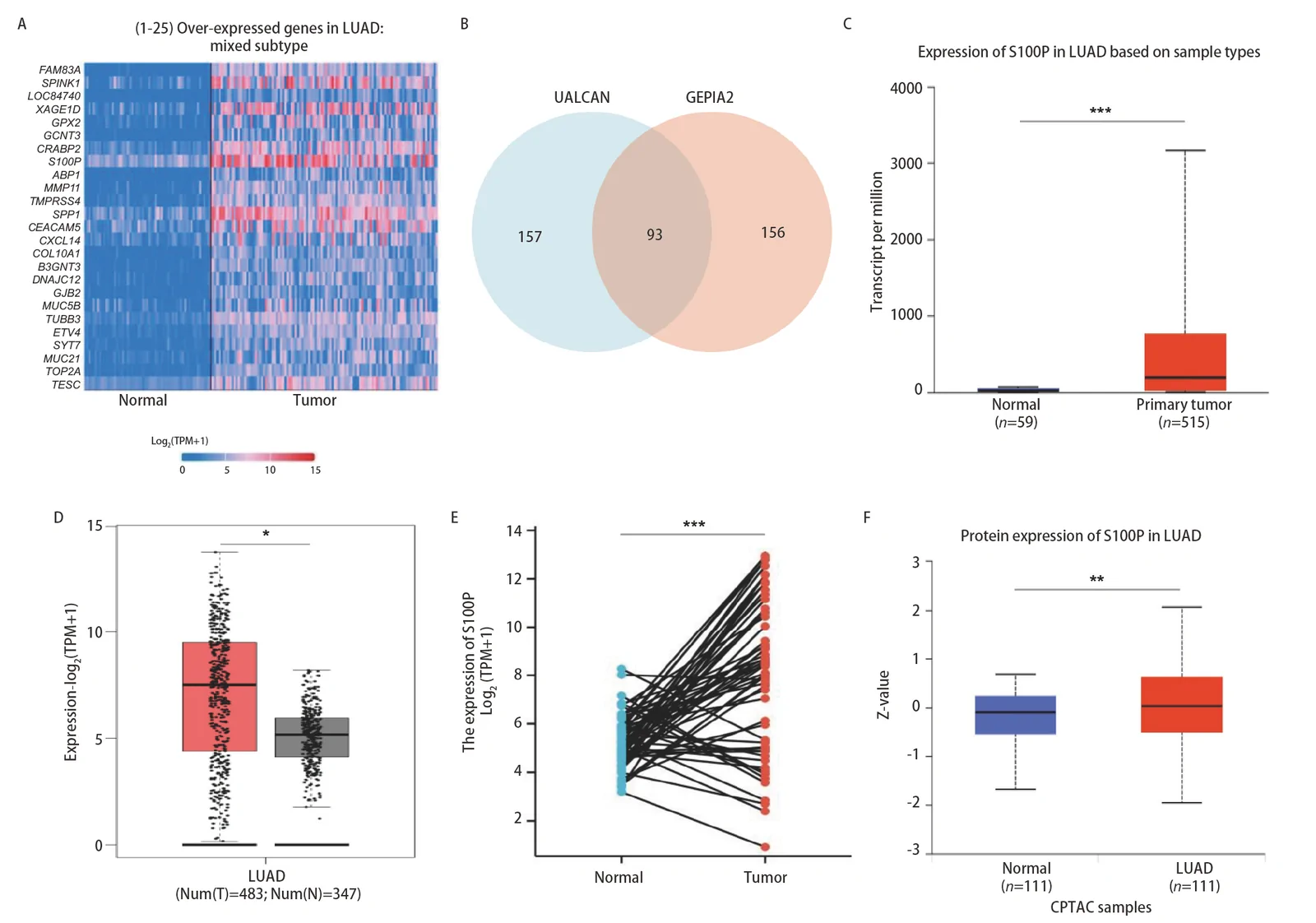
S100P在LUAD中高表达。A：UALCAN数据库中LUAD高表达基因（1-25）热图；B：UALCAN、GEPIA2数据库LUAD前250位高表达基因韦恩图；C-E：S100P在正常与LUAD组织中的mRNA表达（UALCAN数据库、GEPIA2数据库和TCGA-LUAD数据集）；F：S100P在正常与LUAD组织中的蛋白表达（UALCAN数据库）。

在UALCAN数据库泛癌分析发现S100P基因在LUAD、乳腺癌、宫颈癌等肿瘤中高表达（图2A），同时在UALCAN数据库、GEPIA2数据库和Kaplan-Meier plotter数据库中，LUAD S100P高表达组预后均差于低表达组（图2B-2E），差异具有统计学意义。以上结果提示，S100P mRNA在LUAD中高表达且与不良预后有关，是LUAD的潜在生物标志物。

**图 2:**
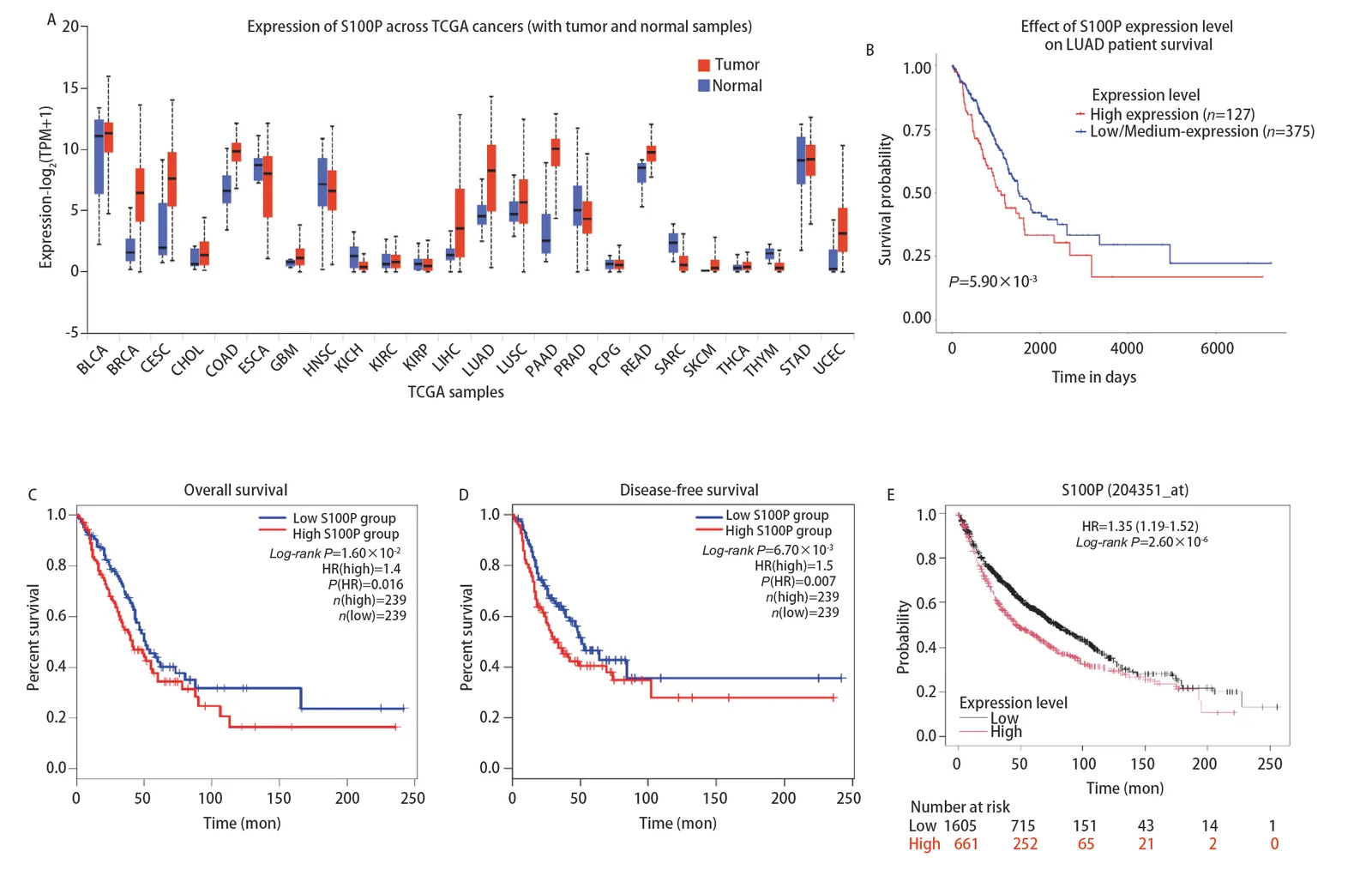
S100P在多种肿瘤中高表达且与LUAD不良预后相关。A：S100P在泛癌中的表达（UALCAN数据库）；B-E：在UALCAN（B）、GEPIA2（C、D）和 Kaplan-Meier plotter（E）数据库中，S100P高表达组LUAD患者预后均差于低表达组。

### 2.2 LUAD中S100P mRNA表达与部分临床特征有关

与正常组相比，男性和女性LUAD患者S100P表达均升高（P=1.62×10^-12^, P=1.99×10^-13^），且男性患者S100P表达高于女性患者（P=1.56×10^-3^），差异均具有统计学意义（图3A）。41-60、61-80、81-100岁三组LUAD患者S100P表达高于正常组（P=3.01×10^-9^, P=1.62×10^-12^, P=5.82×10^-3^），差异具有统计学意义（图3B）。不吸烟与吸烟LUAD患者S100P表达均高于正常组（P均<0.01），差异具有统计学意义（图3C），但不吸烟与吸烟LUAD患者间S100P表达未见统计学意义的差异（P均>0.05）。而各临床分期LUAD患者S100P表达均高于正常组（P均<0.01），差异具有统计学意义（图3D），但各临床分期LUAD患者间S100P表达未见具有统计学意义的差异（P均>0.05）。以上结果表明，LUAD患者S100P mRNA表达可能与性别、年龄有关，但与是否吸烟和临床分期无关联。

**图 3:**
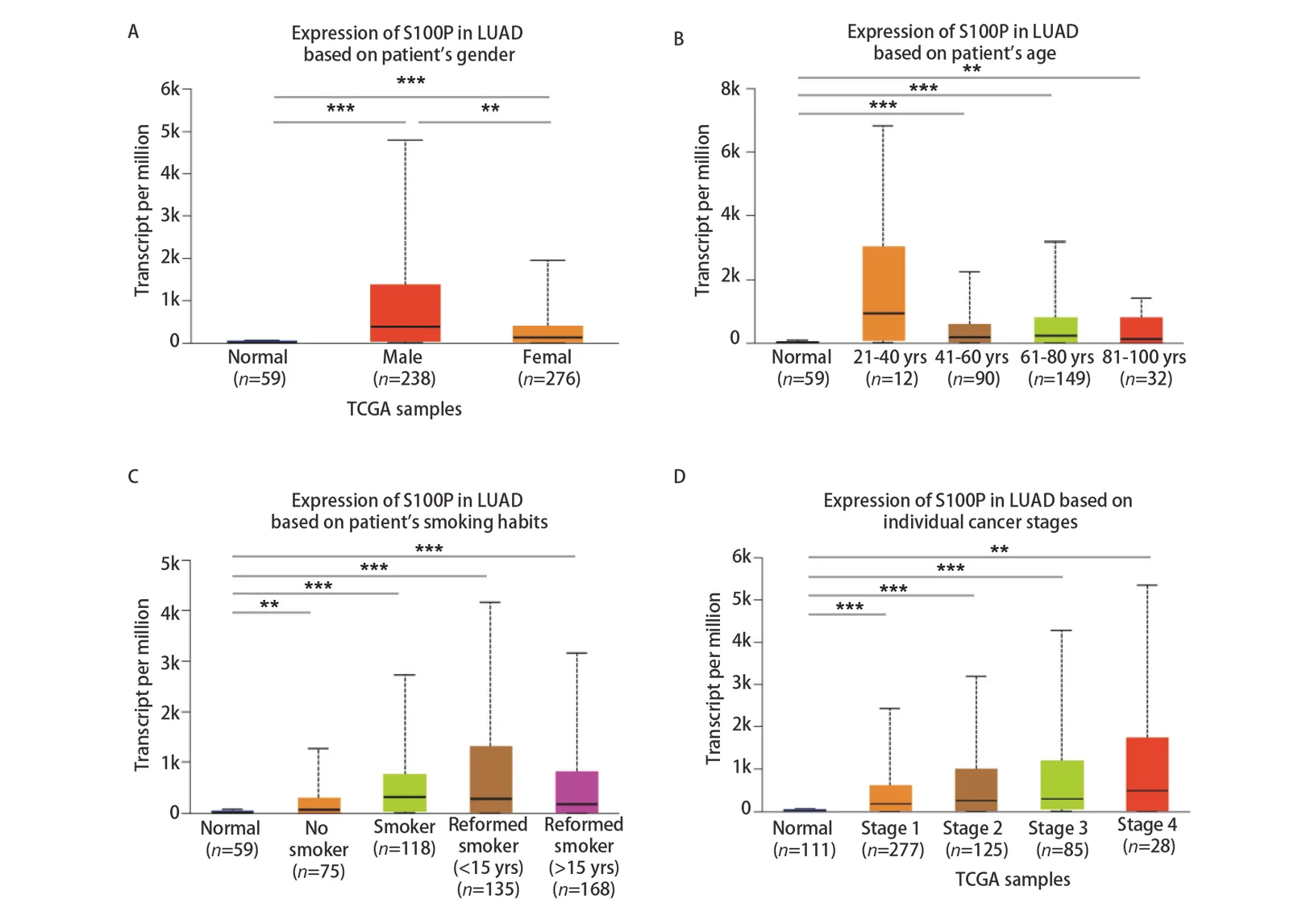
UALCAN数据库中LUAD S100P表达与患者临床特征的相关性。A：S100P在不同性别LUAD患者中的表达；B：S100P在不同年龄LUAD患者中的表达；C：S100P在不同吸烟状态LUAD患者中的表达；D：S100P不同临床分期LUAD患者中的表达。

### 2.3 LUAD中S100P表达与淋巴细胞浸润有关

分别利用获取自UALCAN数据库和GEPIA2数据库的LUAD高表达基因谱排名前250位的基因做GO/KEGG分析。基于UALCAN数据库LUAD高表达基因谱的GO/KEGG分析结果显示，高表达基因的生物过程（biological process, BP）主要富集于细胞周期的正向调控、淋巴细胞分化的调控、B细胞分化的调控和T细胞分化的调控等，且与细胞周期和p53信号通路相关（图4A）。同时基于GEPIA2数据库的分析结果显示，高表达基因的BP主要富集于B细胞受体信号通路、免疫反应激活细胞表面受体信号通路、淋巴细胞活化正向调节、细胞激活的正向调节等，且也与细胞周期和p53信号通路相关（图4B）。因此，进一步利用GSCA数据库分析S100P mRNA表达与LUAD免疫细胞浸润的关系。结果显示，在LUAD中S100P的表达与CD8^+ ^T细胞、耗竭细胞、中央记忆细胞、B细胞、nTreg细胞、效应记忆细胞和Th17呈正相关，且具有统计学意义（图4C）。进一步基于TIMER3数据库进行了S100P表达预测ICIs疗效的在线分析，受试者工作特征（receiver operating characteristic, ROC）曲线结果显示S100P表达预测LUAD ICIs疗效的曲线下面积（area under the curve, AUC）可达到0.669（图4D）。

**图 4:**
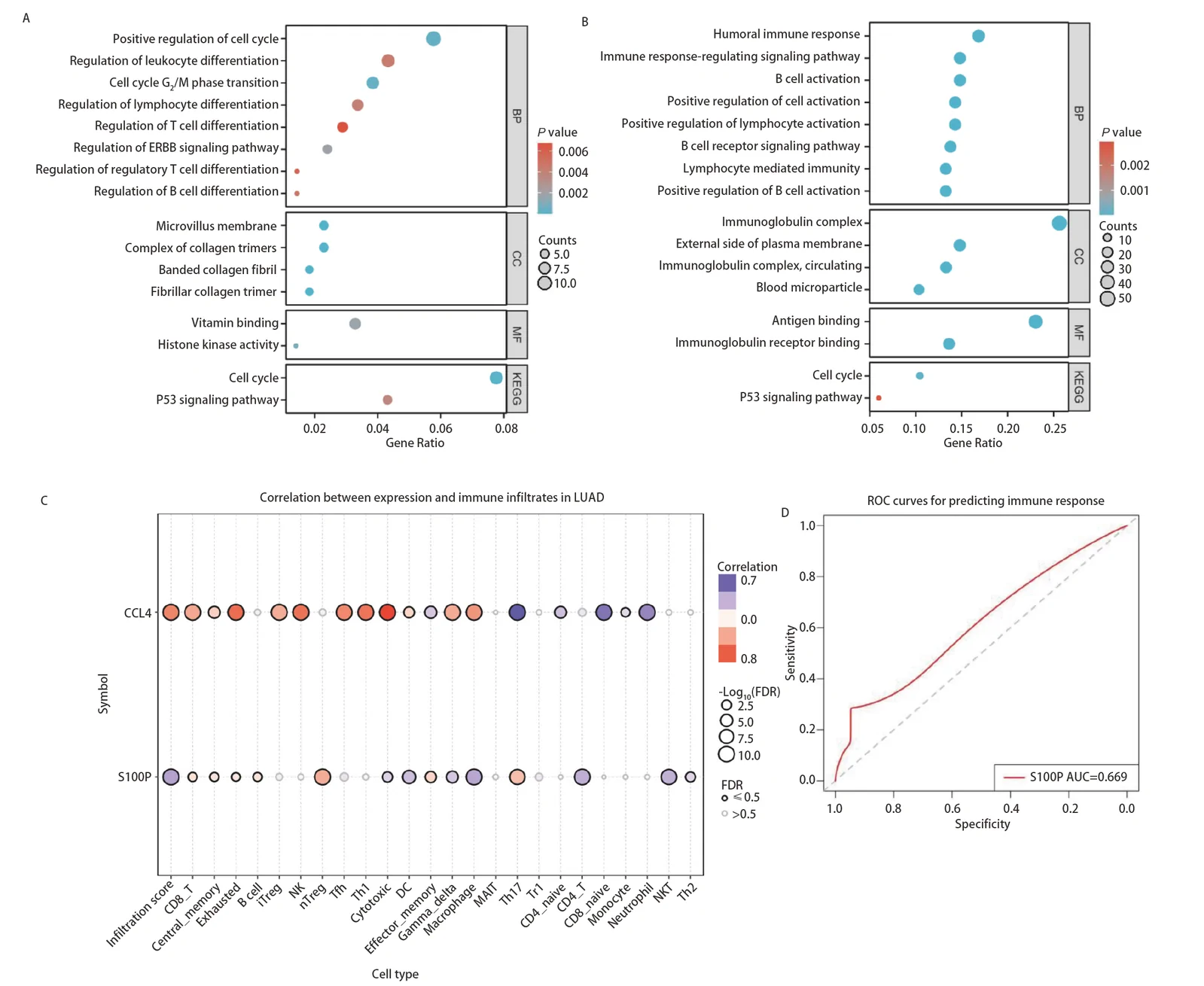
LUAD中S100P表达与免疫相关分析。A：UALCAN数据库LUAD高表达基因谱GO/KEGG分析结果；B：GEPIA2数据库LUAD高表达基因谱GO/KEGG分析结果；C：S100P mRNA高表达与LUAD免疫细胞浸润的关系（GSCA数据库）；D：S100P表达预测LUAD免疫检查点抑制剂疗效（TIMER3数据库）。

同时本研究进一步在GSCA数据库分析了CD8^+ ^T细胞相关趋化因子CCL4与LUAD免疫细胞浸润的关系。结果显示，CCL4也与LUAD CD8^+ ^T细胞、耗竭细胞、中央记忆细胞等免疫细胞的浸润呈正相关且具有统计学意义（图4C）。

### 2.4 S100P蛋白在临床LUAD组织样本高表达

为进一步验证以上生信分析的结果，研究收集了临床LUAD与肺炎症性病变患者病理诊断剩余组织样本，利用免疫组化检测S100P蛋白在LUAD组、正常组（正常癌旁组织）和良性组（炎症性病变）组织中的表达。结果显示，S100P蛋白主要定位于癌细胞胞核和胞浆（图5A-5C），S100P蛋白在LUAD组中的表达7.00（4.00, 9.00）显著高于正常组0.00（0.00, 2.00）和良性组1.00（0.00, 2.00）（图5D）。三组间S100P蛋白表达差异经Kruskal-Wallis H检验具有统计学意义（H=82.164, P=1.44 ×10^-18^）后采用Dunn’s test进行两两比较，并经过Bonferroni校正，结果显示LUAD组与良性组（P=4.05×10^-13^），LUAD组与正常组（P=5.50×10^-16^）之间的蛋白表达差异具有统计学意义。而良性组与正常组之间差异不具有统计学意义（P=0.999）。LUAD组织中S100P蛋白呈轻、中和重度阳性表达的比例分别为30.00%、20.00%和46.00%，阳性表达总体发生率为96.00%，高于正常组（28.00%）及良性组（36.00%）。提示S100P蛋白在LUAD组织中也呈整体上调趋势，其差异主要体现于表达强度而非单纯阳性率。

**图 5:**
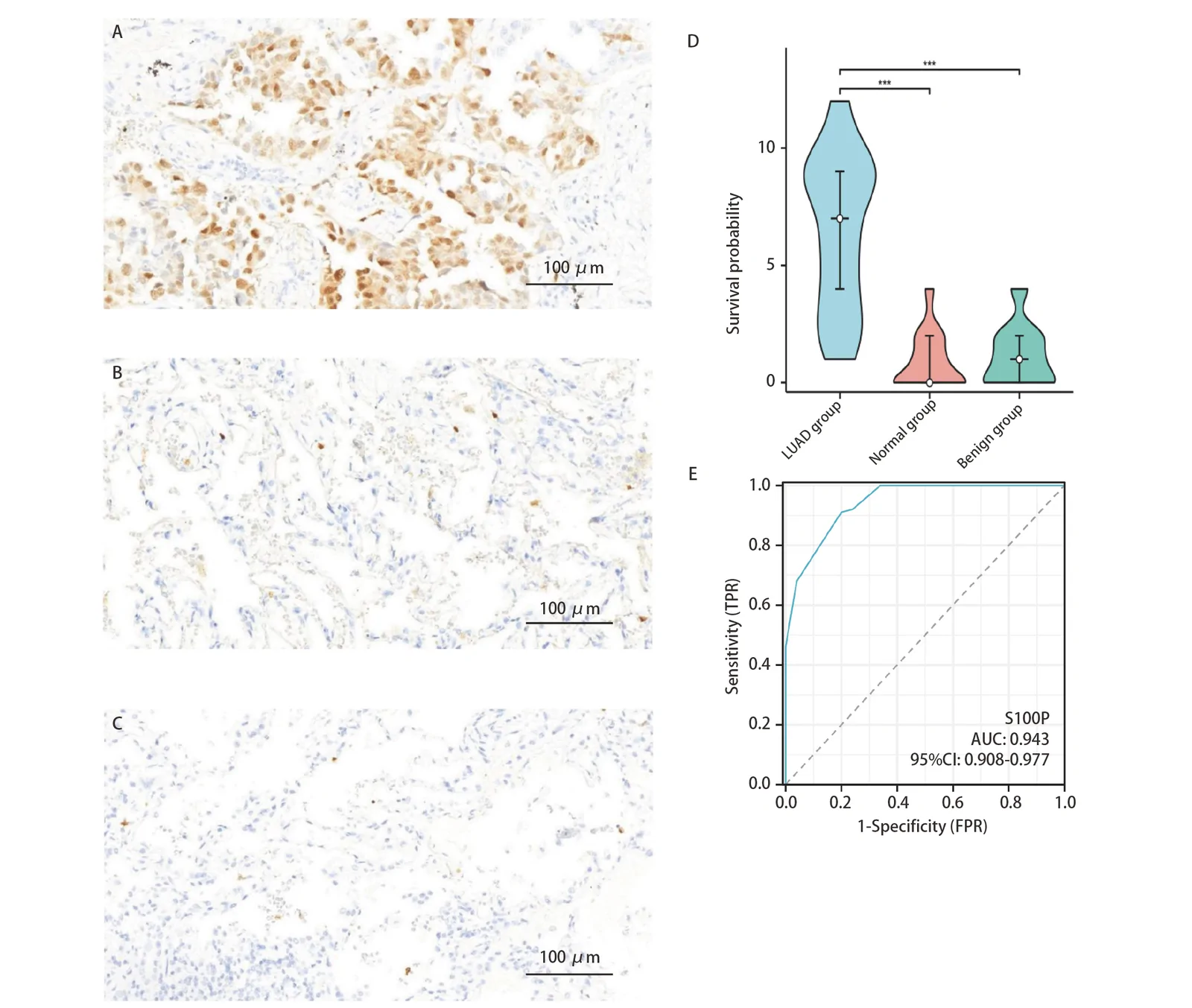
S100P蛋白在临床组织样本中的表达（SP法，×200）。A-C：S100P蛋白在临床LUAD组（A）、正常组（B）和良性组（C）中的表达；D：S100P蛋白在LUAD组、正常组和良性组肺组织中的免疫组化评分；E：S100P蛋白表达鉴别LUAD的ROC曲线。

将正常组和良性组合并为良性对照组，利用S100P蛋白表达数据进行ROC曲线分析，通过约登指数法确定最佳截断值。可见S100P蛋白表达用于辅助鉴别LUAD AUC可达0.943，最佳截断值为免疫组化评分2.50，此时诊断的灵敏度为91.00%、特异性为80.00%、阳性预测值为90.10%、阴性预测值为81.63%、约登指数为0.71，具有良好的鉴别性能（图5E）。

### 2.5 S100P蛋白表达与LUAD患者临床病理特征的关系

S100P蛋白在癌组织中的表达与LUAD患者的年龄、性别、肿瘤大小及分化程度均无关联（P均>0.05）（表1）。

**表 1:** S100P基因编码蛋白表达与LUAD患者临床病理特征关系

Variable	n	S100P protein		P
		Low expression (n=17)	High expression (n=33)	
Sex				0.480
Male	23	9 (52.94%)	14 (42.42%)	
Female	27	8 (47.06%)	19 (57.58%)	
Age (yrs)				0.903
≤60	20	7 (41.18)	13 (39.39%)	
>60	30	10 (58.82%)	20 (60.61%)	
Tumor size (cm)				0.419
≤3	42	13 (76.47%)	29 (87.88%)	
>3	8	4 (23.53%)	4 (12.12%)	
Differentiation				0.277
Moderately differentiated	40	12 (70.59%)	28 (84.85%)	
Poorly differentiated	10	5 (29.41%)	5 (15.15%)	

### 2.6 CCL4和CD8蛋白在LUAD组织样本高表达——免疫组化检测S100P相关分子CCL4和CD8蛋白在LUAD组、正常组和良性组组织中的表达

CCL4蛋白主要定位于癌细胞胞浆（图6A-6C），CD8蛋白主要定位于浸润淋巴细胞胞膜（图6D-6F ），图中两种抗体均呈棕黄色染色。

**图 6:**
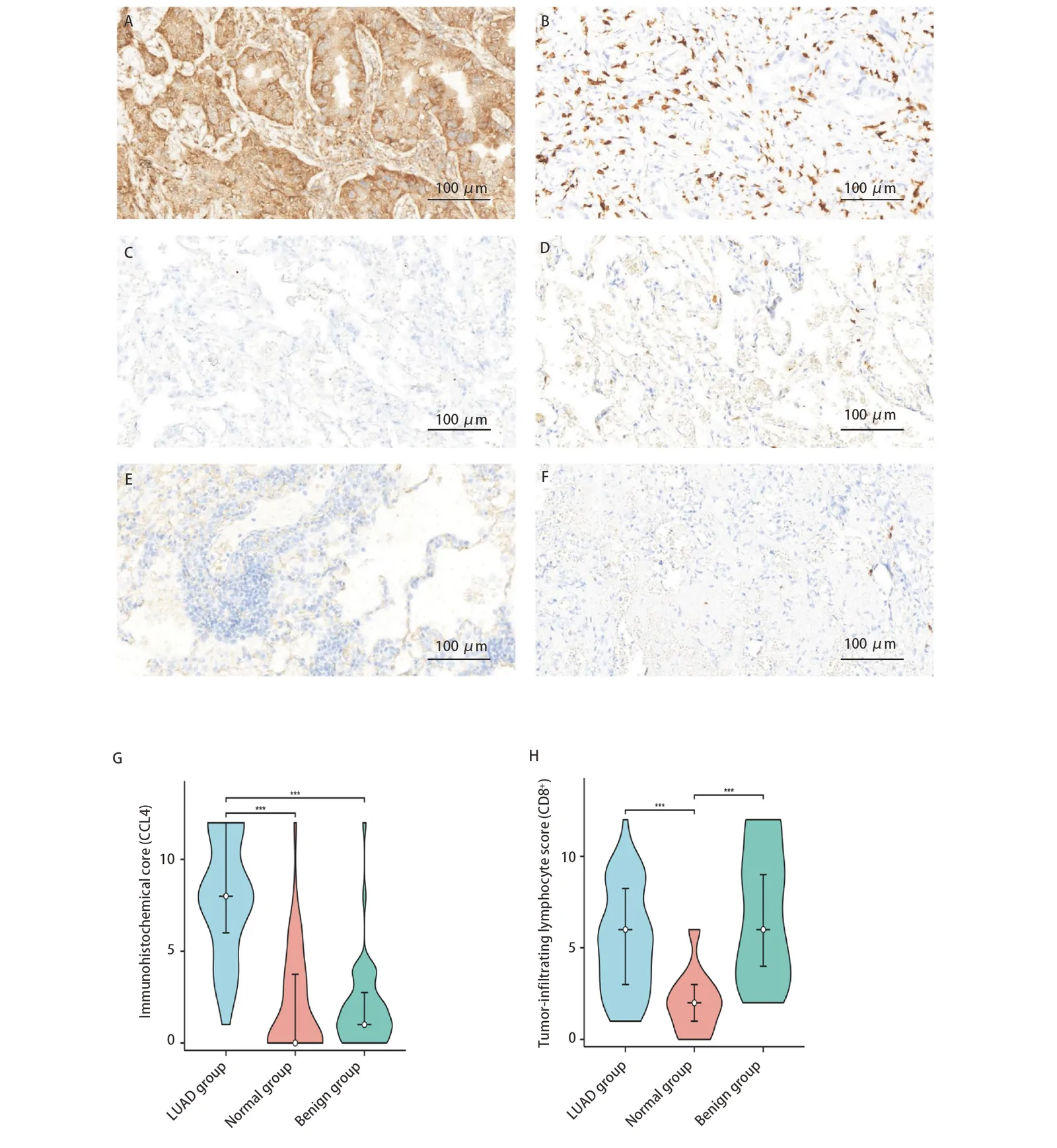
CCL4、CD8蛋白在临床组织样本中的表达（SP法，×200）。A-C：CCL4蛋白在LUAD组（A）、正常组（B）和良性组肺组织（C）中的表达； D-F：CD8^+^免疫细胞在LUAD组（D）、正常组（E）和良性组肺组织（F）中的浸润情况；G：CCL4蛋白在LUAD组、正常组和良性组肺组织中的免疫组化评分；H：LUAD组、正常组和良性组中CD8^+^免疫细胞的肿瘤浸润淋巴细胞评分。

CCL4蛋白在LUAD组中的表达8.00（6.00, 12.00）显著高于正常组0.00（0.00, 3.75）及良性组1.00（1.00, 2.75）（图6G），三组间CCL4蛋白表达差异经Kruskal-Wallis H检验具有统计学意义（H=74.096, P=8.13×10^-17^），后采用Dunn’s test进行两两比较，并经过Bonferroni校正，结果显示LUAD组与良性组（P=1.89×10^-12^）、LUAD组与正常组（P=4.39×10^-14^）之间的CCL4蛋白表达差异具有统计学意义；而良性组与正常组之间的差异不具有统计学意义（P=0.999）。LUAD组、正常组及良性组组织的CCL4蛋白阳性率分别为80.00%、42.00%、48.00%。

LUAD组组织中CD8^+^ T细胞浸润程度较正常组显著升高。LUAD组CD8^+^ T细胞浸润评分为6.00（3.00, 8.25），正常组为2.00（1.00, 3.00），良性组为6.00（4.00, 9.00）。三组间差异经Kruskal-Wallis H检验具有统计学意义（H=58.45, P=2.03×10^-13^）后采用Dunn’s test进行两两比较，并经过Bonferroni校正，结果显示正常组与良性组（P=8.76×10^-13^），LUAD组与正常组之间的差异显著（P=5.69×10^-8^）；而良性组与LUAD组之间差异不显著（P=0.28）（图6H）。LUAD组、正常组及良性组的阳性率分别为82.00%、62.00%、100.00%。

### 2.7 S100P、CCL4蛋白表达和CD8^+^ T细胞浸润正相关

去除良性组（炎症性病变），在LUAD组和正常组组织样本中，S100P与CCL4的蛋白表达呈中度正相关（r= 0.611, P<0.001）（图7A）；进一步分析发现，S100P蛋白的高表达同样与CD8^+^ T细胞浸润水平呈正相关（r=0.461, P<0.001）（图7B）；相应地，CCL4蛋白的表达与CD8^+ ^T细胞浸润也呈正相关（r=0.400, P<0.001）（图7C）。提示S100P可能通过促进CCL4的分泌而增强效应性T细胞募集。

**图 7:**
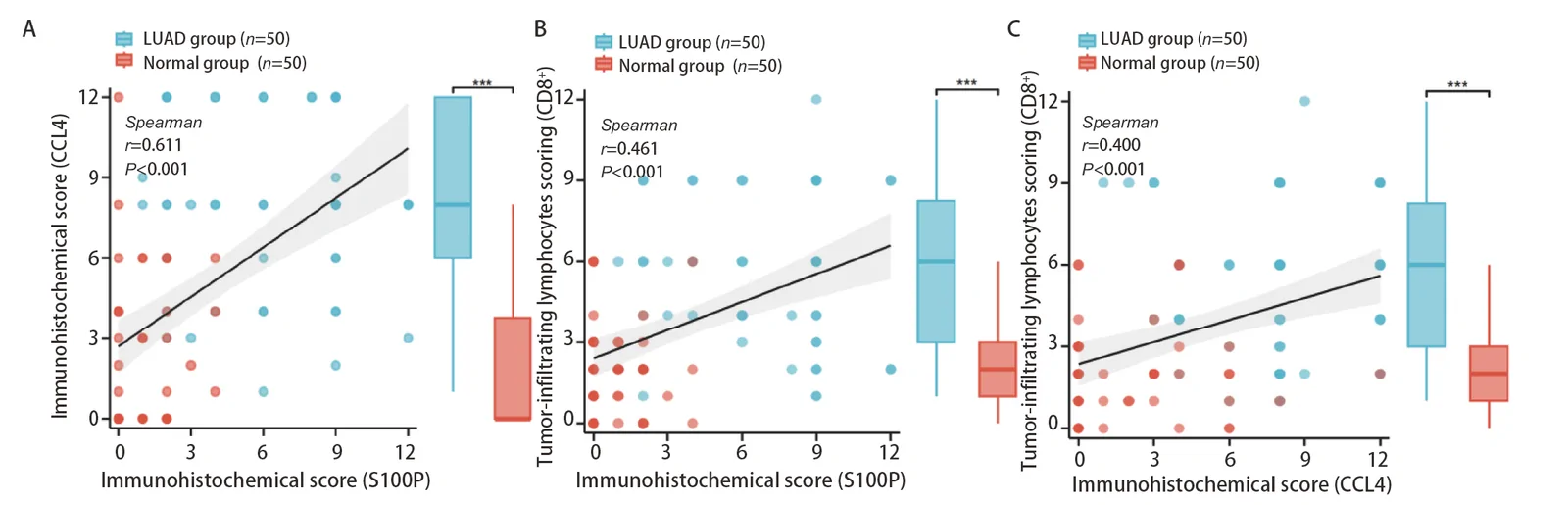
S100P、CCL4、CD8^+^蛋白表达相关性分析。A：S100P与CCL4的蛋白表达相关性分析；B：S100P蛋白表达与CD8^+^免疫细胞浸润的相关性分析；C：CCL4蛋白表达与CD8^+^免疫细胞浸润的相关性分析。

## 3 讨论

在我国，肺癌是最常见的恶性肿瘤之一，其发病率与病死率均位居首位^[7]^。与大多数西方国家的肺癌发病率相比，中国的肺癌发病率仍在不断上升^[8]^。LUAD患者往往处于中、晚期时被确诊，预后较差，总体生存率较低。因此，探究LUAD的特异性标志物对于肺癌的早期诊疗及预后评估至关重要。

S100P属于S100钙结合蛋白家族成员之一，具有典型的EF-hand结构域，能够与Ca²^+^结合并介导多种信号转导过程。研究^[9]^表明，S100P既可通过胞外分泌与受体RAGE结合，激活下游丝裂原活化蛋白激酶（mitogen-activated protein kinase, MAPK）/细胞外信号调节激酶（extracellular signal-regulated kinase, ERK）、核转录因子-κB（nuclear factor-κB, NF-κB）及Janus激酶/信号转导和转录活化因子（Janus kinase/signal transducer and activator of transcription, JAK/STAT）等经典信号通路，促进细胞增殖、迁移及抗凋亡反应；亦可通过与Ezrin及IQGAP1等细胞骨架相关蛋白结合，调控细胞黏附及骨架重塑，诱导上皮-间质转化（epithelial-mesenchymal transition, EMT），进一步增强肿瘤细胞的侵袭与转移潜能。综合来看，S100P通过RAGE依赖性受体信号及多靶蛋白介导的胞内信号网络，在多种肿瘤的发生发展中发挥重要的促癌作用。目前的研究证实S100P在多种癌症中过度表达，如胃癌^[10]^、胆囊癌^[11]^、结直肠癌^[9]^及乳腺癌^[9]^等，其表达水平的异常改变在肿瘤发生发展进程中发挥着关键作用，与多种癌症的异常增殖、浸润和转移密切相关，本研究通过数据库分析亦证实这一点。同时本研究通过数据库分析LUAD患者S100P mRNA表达与性别和年龄存在关联，而与是否吸烟和临床分期无明显关联，但通过免疫组化检测发现S100P蛋白水平表达与性别和年龄不存在相关性，提示可能S100P受年龄、性别影响的转录调控较弱，但蛋白稳定性无明显差异；且通过免疫组化检测发现S100P在LUAD组织中的蛋白表达水平明显高于正常癌旁组织与炎症病变组织，提示S100P与LUAD的发生发展有着密切关联。但是目前关于S100P在肺癌肿瘤微环境（tumor microenvironment, TME）中的具体作用机制研究仍较少。近年来，TME被认为是决定肿瘤进展、免疫逃逸与治疗应答的关键。TME中除肿瘤细胞外，还包含成纤维细胞、血管内皮细胞、免疫细胞（例如CD8^+^ T细胞、肿瘤相关巨噬细胞）和趋化因子等。这些成分相互作用、共同影响肿瘤的生物学行为。已有文献^[12]^指出，TME中的免疫细胞浸润受多种细胞因子和趋化因子调控，其中CCL4是调节免疫细胞募集的重要趋化因子，可通过与其受体CCR5结合，诱导CCR5^+^ T细胞（尤其是CD8^+^ T细胞）向肿瘤区域聚集，从而增强抗肿瘤免疫反应。此外，已有研究^[13]^显示，NF-κB可广泛调控包括CCL4在内的趋化因子和细胞因子基因的转录表达，而S100P正是NF-κB经典激活因子的上游调控因子之一。但是目前尚无实验验证S100P调控CCL4的机制，仅有炎症细胞（如单核细胞）模型及机制研究支持NF-κB对CCL4的调控能力。因此推测S100P可能通过激活RAGE→NF-κB信号通路上调CCL4水平，从而增强CD8^+ ^T细胞在肿瘤组织中的募集和活化，参与抗肿瘤免疫反应，有待进一步验证。

基于此，本研究首先分别利用获取自UALCAN数据库和GEPIA2数据库的LUAD高表达基因谱排名前250位的基因做GO/KEGG分析，研究发现来自2个数据库的LUAD高表达基因谱生物功能均可富集到免疫细胞的活化与分化上，提示LUAD可能与TME中免疫细胞的富集与活化有关。而S100P作为LUAD高表达基因谱排名前10的差异基因代表，其可能也与TME中淋巴细胞的富集与活化有关，并提示S100P mRNA表达与LUAD免疫细胞浸润正相关，是一个潜在的LUAD免疫标志物。同时发现S100P与CCL4 mRNA表达均与LUAD多种免疫细胞浸润相关，特别是CD8^+ ^T细胞。

本研究进一步使用免疫组化的方法检测了肿瘤和癌旁组织中CCL4及CD8的蛋白表达水平，以此验证S100P、CCL4及CD8在LUAD发生发展中的调节作用。实验结果亦显示S100P、CCL4在肿瘤组织的表达均明显高于正常癌旁组织及炎症病变组织，CD8在肿瘤组织的表达明显高于正常癌旁组织；并且三者表达水平具有相关性，均呈中度正相关。因此，本实验观察发现在LUAD中S100P表达上调，同时CCL4水平也上调，并且能够介导CD8^+^ T细胞募集，细胞功能处于耗竭状态，可能具有增强抗肿瘤免疫作用的趋势，同时，在炎症病变组中，由于病毒、细菌等病原体感染肺组织，抗原呈递细胞将病原体抗原呈递给CD8细胞，也使其增殖、活化；本实验亦提示CD8表达升高，与肺癌组无显著差异，代表正常的抗免疫应答，CD8细胞功能正常。但S100P上调CCL4表达的信号通路，以及CCL4被上调后影响肺癌TME中免疫细胞招募、活化的具体机制，包括对T细胞、自然杀伤细胞等免疫细胞功能的调控机制均尚不明确，仍需进行进一步的实验验证以及更深入的研究探讨，这也是后续的研究重点。

本研究具有一定的局限性，具体为样本量相对有限，可能影响统计学效能及结论的普遍性。且病例来源、组织学分级及分子分型可能对结果产生影响，相关结论仍需在更大样本及多中心研究中进一步验证；且关于S100P预后价值的证据主要来源于公共数据库生存分析，而本院临床队列尚缺乏完整的随访资料，未能进一步结合总生存期、无病生存期或复发情况进行验证，也未开展多因素分析以判断S100P是否为独立预后因素。

综上所述，本研究表明，S100P和CCL4的表达与肺癌的发生发展密切相关，有望成为肺癌早期诊断的新型生物标志物，提高肺癌早期诊断的准确性，有助于实现肺癌的早发现、早诊断、早治疗。另外，基于S100P上调CCL4能够介导CD8^+^ T细胞募集从而增强抗肿瘤免疫的作用，可以开发针对该调节通路的新型治疗策略，为LUAD治疗提供了新靶点。本研究将进一步扩大样本量进行分析，深入研究S100P上调CCL4表达的信号通路，将为今后了解肿瘤发生发展的分子机制提供重要的帮助，也将为肿瘤的治疗提供新的理论依据和选择方向。
